# Visual ecology of the Australian lungfish (*Neoceratodus forsteri*)

**DOI:** 10.1186/1472-6785-8-21

**Published:** 2008-12-18

**Authors:** Nathan S Hart, Helena J Bailes, Misha Vorobyev, N Justin Marshall, Shaun P Collin

**Affiliations:** 1 School of Biomedical Sciences, The University of Queensland, Brisbane, QLD 4072, Australia; 2 Faculty of Life Sciences, The University of Manchester, Oxford Road, Manchester, M13 9PL, UK; 3 Department of Optometry and Vision Science, The University of Auckland, Private Bag 92019, Auckland, New Zealand

## Abstract

**Background:**

The transition from water to land was a key event in the evolution of vertebrates that occurred over a period of 15–20 million years towards the end of the Devonian. Tetrapods, including all land-living vertebrates, are thought to have evolved from lobe-finned (sarcopterygian) fish that developed adaptations for an amphibious existence. However, while many of the biomechanical and physiological modifications necessary to achieve this feat have been studied in detail, little is known about the sensory adaptations accompanying this transition. In this study, we investigated the visual system and visual ecology of the Australian lungfish *Neoceratodus forsteri*, which is the most primitive of all the lungfish and possibly the closest living relative to the ancestors of tetrapods.

**Results:**

Juvenile *Neoceratodus *have five spectrally distinct retinal visual pigments. A single type of rod photoreceptor contains a visual pigment with a wavelength of maximum absorbance (λ_max_) at 540 nm. Four spectrally distinct single cone photoreceptors contain visual pigments with λ_max _at 366 (UVS), 479 (SWS), 558 (MWS) and 623 nm (LWS). No double cones were found. Adult lungfish do not possess UVS cones and, unlike juveniles, have ocular media that prevent ultraviolet light from reaching the retina. Yellow ellipsoidal/paraboloidal pigments in the MWS cones and red oil droplets in the LWS cones narrow the spectral sensitivity functions of these photoreceptors and shift their peak sensitivity to 584 nm and 656 nm, respectively. Modelling of the effects of these intracellular spectral filters on the photoreceptor colour space of *Neoceratodus *suggests that they enhance their ability to discriminate objects, such as plants and other lungfishes, on the basis of colour.

**Conclusion:**

The presence of a complex colour vision system based on multiple cone types and intracellular spectral filters in lungfishes suggests that many of the ocular characteristics seen in terrestrial or secondarily aquatic vertebrates, such as birds and turtles, may have evolved in shallow water prior to the transition onto land. Moreover, the benefits of spectral filters for colour discrimination apply equally to purely aquatic species as well as semi-aquatic and terrestrial animals. The visual system of the Australian lungfish resembles that of terrestrial vertebrates far more closely than that of other sarcopterygian fish. This supports the idea that lungfishes, and not the coelacanth, are the closest living relatives of the ancestors of tetrapods.

## Background

Lungfishes (Dipnoi) diverged from the main vertebrate stock in the early Devonian (ca. 390 MYA) [1] and together with coelancanths and the extinct osteolepimorphs comprise the Sarcopterygii (lobe-finned fish). While it is generally agreed that tetrapods evolved from sarcopterygian fish, phylogenetic relationships within the Sarcopterygii have been the subject of considerable debate [2,3]. A recent consensus, based on the analysis of nuclear DNA [4] mitochondrial DNA [5] and ribosomal RNA gene sequences [6], is that lungfishes rather than coelacanths are the closest living relatives of the tetrapods.

**Figure 1:**
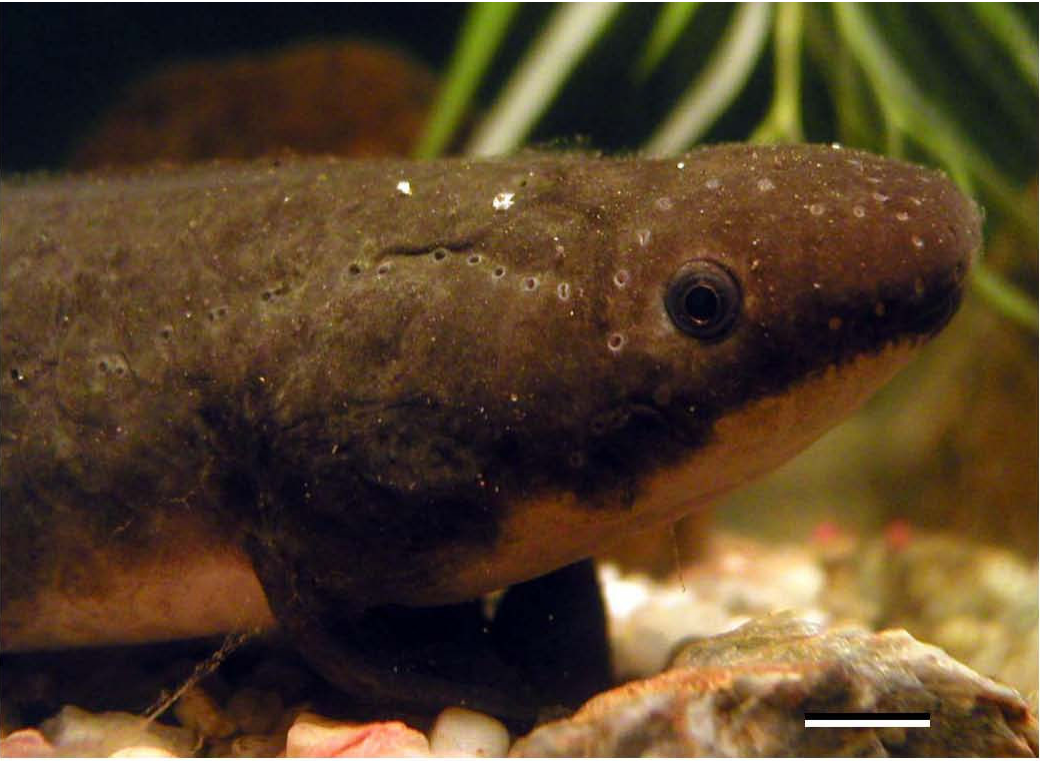
**A juvenile (TL = 25 cm) Australian lungfish, *Neoceratodus forsteri***. Note the strong dorso-ventral countershading and pale belly colouration. Scale bar = 1 cm. This photograph, taken by HJB and NJM, appeared as a cover image in the British Journal of Ophthalmology (Vol. 90, number 7) and is reproduced with kind permission from the BMJ Publishing Group.

Worldwide, there are six extant species of lungfishes: four in Africa, one in South America and one in Australia. All African (*Protopterus *sp.) and South American (*Lepidosiren*) lungfishes belong to the Lepidosirenidae; the Australian lungfish (*Neoceratodus forsteri*; Fig. 1) is the only surviving species in the Ceratodontidae [4,7]. Compared with the other species of lungfishes, which have paired lungs, *Neoceratodus *has a single lung and uses predominantly gill respiration, only breathing air during periods of increased activity or when water quality is poor [7]. Based on this and other morphological, molecular and palaeontological evidence, *Neoceratodus *is considered to be the more primitive lungfish species [8,9].

Although originally considered to be rather degenerate [10] and of little behavioural importance [11], the visual system of the lungfish represents something of a conundrum. The eyes of lungfishes are small relative to body size when compared with other vertebrates [12-14] and have low spatial resolving power [15,16]. Moreover, behavioural studies of both captive and wild populations of *Neoceratodus *suggest that, except during the breeding season, they are predominantly crepuscular or nocturnal in habit [7,17]. Nevertheless, the retina of *Neoceratodus *has at least three morphologically distinct cone photoreceptor types, some of which contain brightly coloured oil droplets or spectral filters [15,18], and expresses multiple visual pigment opsin genes [19]. Coloured oil droplets/spectral filters (combined with multiple spectral subtypes of cone photoreceptor) are characteristic of strongly diurnal (and generally terrestrial) non-mammalian vertebrates, such as birds, turtles and lizards [10] and are thought to improve colour vision by tuning the spectral sensitivity functions of the cones [20,21]; densely pigmented spectral filters are generally absent from nocturnal or crepuscular species [10] because they absorb light and reduce absolute sensitivity.

Thus, the polychromatic visual system of the lungfish is at odds with its known behavioural characteristics and raises a number of interesting questions with regard to the neuroethology of the lungfish, the function and utility of coloured oil droplets and the evolution of the tetrapod visual system. To investigate these issues, we have measured some of the most important parameters of the visual ecology of *Neoceratodus forsteri*: the spectral absorption characteristics of the visual pigments and spectral filters (pigmented oil droplets and diffuse pigment in the ellipsoid and paraboloid regions) of the retinal photoreceptors, the spectral transmittance of the ocular media, the spectral distribution of the ambient light in a typical lungfish habitat and the spectral reflectance of relevant objects in its environment. Using these data, we have modelled the performance of the lungfish visual system with respect to its ability to distinguish both real and theoretical coloured objects.

## Results

### Photoreceptor visual pigments, spectral filters and spectral sensitivity

**Figure 2:**
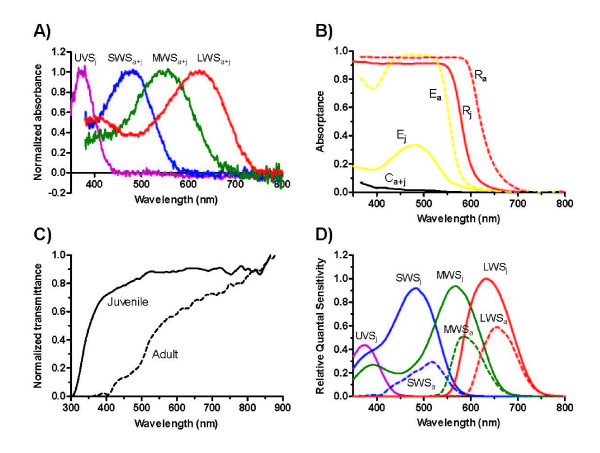
**Spectral characteristics of retinal photoreceptors and ocular media in juvenile and adult Australian lungfish (*Neoceratodus forsteri*)**. **(A) **Normalized absorbance spectra of cone photoreceptor visual pigments. UVS, SWS, MWS and LWS refer to the pigments found in the ultraviolet-, short-, medium- and long-wavelength-sensitive cones, which have wavelengths of maximum absorbance (λ_max_) at 366, 479, 558 and 623 nm, respectively. The absorbance spectrum of the rod pigment (λ_max _540 nm) is not shown. All pigments are found in juvenile lungfish (j), but the UVS cones are absent from adults (a). **(B) **Absorptance spectra of intracellular organelles and spectral filters. R, E and C refer to the red oil droplets, yellow ellipsoid pigment and colourless oil droplets in the LWS, MWS and SWS/UVS cones (spectra pooled), respectively. The absorptance spectrum of the yellow paraboloid pigment in the MWS cones of adult *Neoceratodus *(not shown) is almost identical to that of the ellipsoidal pigment in juveniles. **(C) **Normalized transmittance spectra of the combined ocular media (lens, cornea, etc.) of adult and juvenile lungfish. Note the increased absorption of shorter wavelength light by the ocular media of the adult. **(D) **Calculated quantal spectral sensitivities of the 4 cone types in juveniles and 3 cone types in adults, taking into account the spectral filtering effects of the intracellular spectral filters and ocular media on the absorption of light by the visual pigment in each cone type

The retina of the Australian lungfish is duplex, containing both rod and cone photoreceptors. Microspectrophotometry showed that juvenile lungfish possess four types of single cone photoreceptor with visual pigments maximally sensitive to ultraviolet (UVS, λ_max _366 nm), short- (SWS, λ_max _479 nm), medium- (MWS, λ_max _558 nm) and long-wavelengths (LWS, λ_max _623 nm; Table 1 and Fig. 2A) and, therefore, have the potential for tetrachromatic colour vision. UVS cones are the only photoreceptor type not found in the retina of the adult lungfish. The retinae of both juveniles and adults also contain a single type of MWS rod (λ_max _540 nm). We did not observe double cones in the lungfish retina, a finding that supports previous anatomical studies [15]. On the basis of spectral bandwidth and the fit of the absorbance spectra to visual pigment templates [22], all visual pigments are considered to be porphyropsins (visual pigments utilising the vitamin A_2_-based chromophore, 3,4-didehydroretinal), as is typical of other freshwater fish [23].

**Table 1:** Summary of spectral and morphological characteristics of retinal photoreceptors in juvenile and adult Australian lungfish (*Neoceratodus forsteri*).

	Rods	Cones			
	
		UVS	SWS	MWS	LWS
		
*Visual pigments*					
Mean λ_max _of prebleach spectra (nm)	539.8 ± 1.1	366.3 ± 2.2	479.2 ± 2.9	558.2 ± 4.8	623.2 ± 3.3
λ_max _of mean prebleach spectrum (nm)	539.7	366.5	478.8	558.5	623.6
Mean λ_max _of difference spectra (nm)	542.9 ± 1.4	367.6 ± 2.1	480.5 ± 4.0	558.8 ± 4.4	623.4 ± 2.3
λ_max _of mean difference spectrum (nm)	542.8	367.6	479.1	557.7	623.3
Mean transverse (decadic) absorbance at λ_max _of difference spectrum	0.137 ± 0.029	0.023 ± 0.004	0.023 ± 0.007	0.037 ± 0.010	0.037 ± 0.010
Mean outer segment length (μm)	31.7 ± 4.7	5.2 ± 1.6	8.0 ± 3.3	8.3 ± 1.5	8.3 ± 1.9
Mean outer segment tip diameter (μm)	-	1.8 ± 0.3	2.7 ± 0.9	2.5 ± 0.7	2.8 ± 1.0
Mean outer segment base diameter (μm)	11.1 ± 1.9	3.4 ± 0.7	4.7 ± 1.0	4.1 ± 1.4	5.3 ± 1.8
Number of cells used in analysis (measured)	32 (34)	3 (5)	9 (18)	11 (19)	15 (22)

*Spectral filters*		C	C	E/P	R

Oil droplet diameter (μm)	-	2.8 ± 0.8	4.1 ± 1.9	-	^a^12.6 ± 3.4 ^j^9.1 ± 1.6
Ellipsoid diameter (μm)	-	-	-	^a^5.9 ± 0.7 ^j^6.0 ± 1.4	-
Ellipsoid length (μm)	-	-	-	^a^7.5 ± 1.7 ^j^8.8 ± 2.2	-
Paraboloid diameter (μm)	-	-	-	^a^8.7 ± 1.0	-
Paraboloid length (μm)	-	-	-	^a^9.2 ± 1.3	-
Mean λ_cut _of absorptance spectra (nm)	-	-	-	^Ea^534.3 ± 3.6 ^Ej^503.2 ± 8.8 ^Pa^505.3 ± 4.8	^a^590.8 ± 5.0 ^j^560.1 ± 8.7
λ_cut _of mean absorptance spectrum (nm)	-	-	-	^Ea^534.1 ^Ej^500.5 ^Pa^507.3	^a^590.2 ^j^558.8
Mean λ_mid _of absorptance spectra (nm)	-	-	-	^Ea^555.6 ± 3.1 ^Ej^537.7 ± 17.7 ^Pa^535.4 ± 3.5	^a^622.8 ± 7.3 ^j^581.4 ± 9.9
λ_mid _of mean absorptance spectrum (nm)	-	-	-	^Ea^555.7 ^Ej^534.8 ^Pa^536.3	^a ^622.9 ^j^582.6
Mean maximum transverse absorptance	-	< 0.08	< 0.08	^Ea^097 ± 0.03 ^Ej^0.33 ± 0.05 ^Pa^0.32 ± 0.22	^a^0.96 ± 0.05 ^j^0.94 ± 0.09
Number of cells used in analysis	-	5	17	^Ea^10; ^Ej^8; ^Pa^9	^a^10; ^j^16

Values are ± 1 standard deviation. λ_max_, wavelength of maximum absorbance. The cut-off wavelength (λ_cut_) and wavelength of half maximum measured absorptance (λ_mid_) are supplied for comparison with other microspectrophotometric studies involving densely pigmented spectral filters. The λ_cut _is the wavelength of the intercept at the value of maximum measured absorptance by the line tangent to the absorptance curve at the λ_mid_, as defined by Lipetz [64]. Rods do not contain spectral filters and dimensions are only given for sub-cellular regions that contain spectral filters. C refers to the colourless oil droplets found in the ultraviolet- (UVS, juveniles only) and short-wavelength-sensitive (SWS) single cones. E and P refer to the diffuse yellow pigment located in the ellipsoid and paraboloid (adults only) regions, respectively, of the medium-wavelength-sensitive (MWS) single cones. R refers to the red oil droplets in the LWS single cones. Superscripts ^a ^and ^j ^refer to spectra measured in adult and juvenile lungfish, respectively.

The inner segments of UVS and SWS cones contain one or more colourless oil droplets with minimal absorbance over the measured spectral range (Fig. 2B). MWS cones possess a dense, granular, yellow pigmentation in both the ellipsoid and paraboloid regions of the inner segment that absorbs strongly below 530 nm. A single spherical, ruby red oil droplet occupies the entire width of the ellipsoid of the LWS cones and absorbs all wavelengths below 560 nm. Calculations of spectral sensitivity, taking into consideration the filtering effect of the coloured intracellular spectral filters (Fig. 2B) and the ocular media (Fig. 2C), reveal peak sensitivities for the four cones in juvenile lungfish at 379 nm, 484 nm, 568 nm and 634 nm (Fig. 2D). In adult lungfish, increased absorption of short wavelengths by the ellipsoidal (and paraboloidal) pigments in the MWS cones and the red oil droplet in the LWS cones shifts the peak sensitivities of these photoreceptors to 584 nm and 656 nm, respectively (Fig. 2D). To the best of our knowledge, the calculated peak sensitivity of the adult lungfish LWS cone is the most long-wavelength-shifted of any vertebrate photoreceptor studied to date.

The spectral transmittance characteristics of the ocular media of the juvenile and adult lungfish differ considerably, particularly at short wavelengths (Fig. 2C). The ocular media of the juvenile transmit well across most of the measured spectrum, but absorb strongly below about 390 nm and block all wavelengths below 300 nm. The ocular media of the adult eye appear yellow to the human eye and transmittance decreases gradually as wavelength decreases; light below 400 nm is absorbed completely.

### The effect of spectral filters in lungfish cone photoreceptors on colour discrimination ability

**Figure 3:**
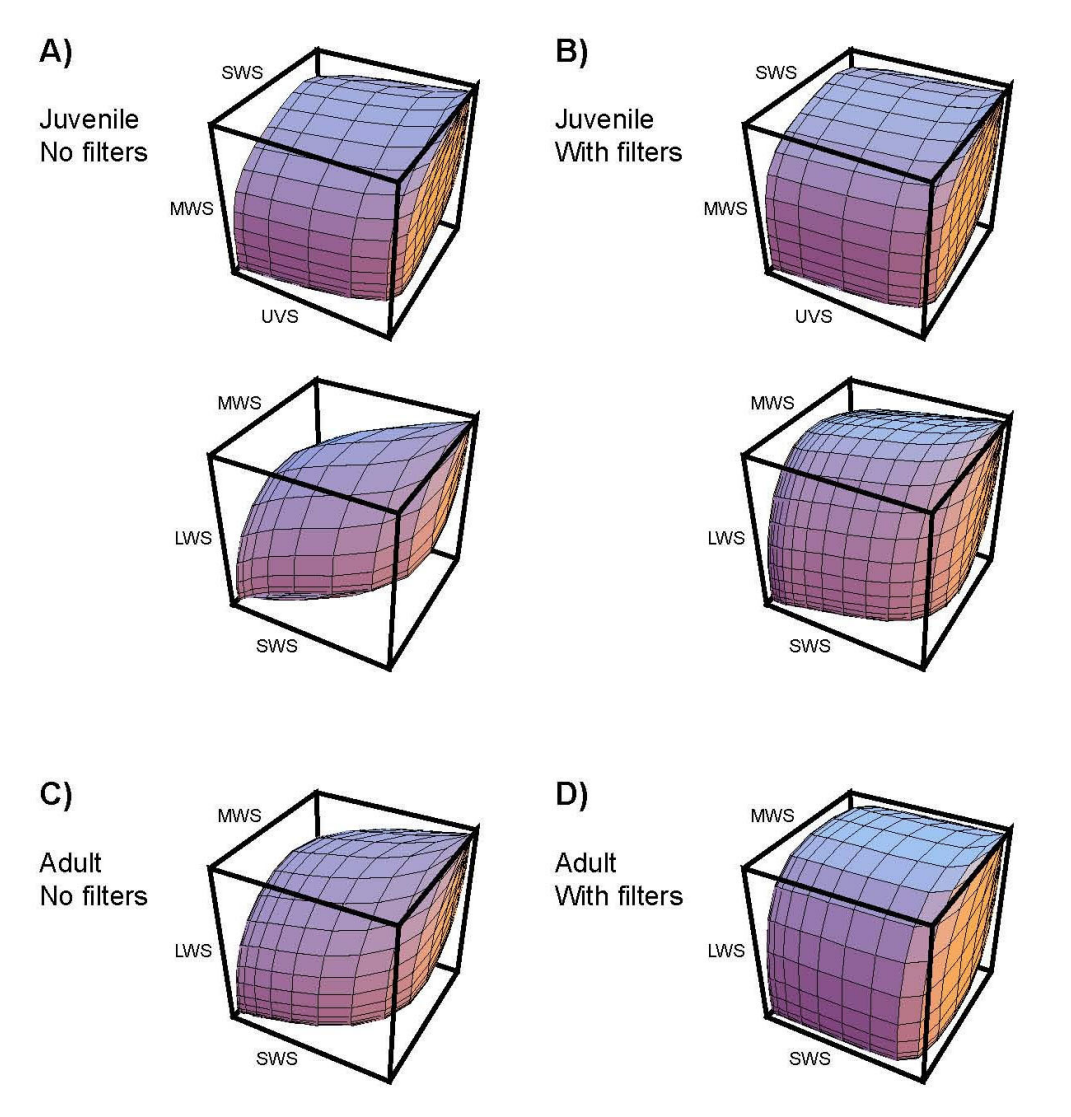
**Object colour solids of juvenile and adult lungfish**. The volume of the object colour solid was calculated for juvenile (**A, B**) and adult (**C, D**) lungfish, both with (**A, C**) and without (**B, D**) the filtering effects of the yellow ellipsoidal and red oil droplet spectral filters in the MWS and LWS cones. The tetrachromatic colour space of the juvenile lungfish (**A, B**) is shown by two different projections of the spaces defined by the quantum catches of the UVS, SWS and MWS cones and also the SWS, MWS and LWS cones. Note the increase in the volume of the object-colour solid (and consequently the number of discriminable colours) in both juveniles (**B**) and adults (**D**) with spectral filters, relative to the situation without the effects of spectral filters. See Fig. 2 for explanation of abbreviations. Projections were generated using Mathematica software (Wolfram Research, Inc., Champaign, IL, USA).

Assuming that the four cone types in the juveniles and the three cones types in the adult subserve tetrachromatic and trichromatic colour vision systems, respectively, we modelled the effect of intracellular spectral filters on the colour vision abilities of the lungfish. To do this, we estimated the total number of theoretical surface colours that the visual systems of both adult and juvenile lungfish can discriminate, with and without spectral filters (Table 2). All surface colours occupy an elementary volume (see Methods) within a mathematical representation of observer colour space known as an 'object-colour solid' [24]. The reduction of overlap between adjacent spectral channels due to the coloured filters is beneficial for colour discrimination, because it increases the volume of object-colour solid [24,25] (Fig. 3). However, the cost of spectral filtering is an increase in signal noise due to the decreased quantum catch of the visual pigment.

**Table 2:** The overall (net) benefit, *r*, of coloured spectral filters* for juvenile and adult lungfish at the surface of the water and at a depth of 1.25 m.

	Juvenile		Adult	
	
	Surface	1.25 m	Surface	1.25 m
*G*	3.74	2.70	4.68	4.45
*G^0^*	1.85	1.50	1.76	1.66
*b*	2.01	1.80	2.64	2.67
*d*	1.54	1.37	2.05	1.98
*r*	1.30	1.31	1.29	1.34

*(yellow ellipsoidal/paraboloidal pigments and red oil droplets)

**Figure 4:**
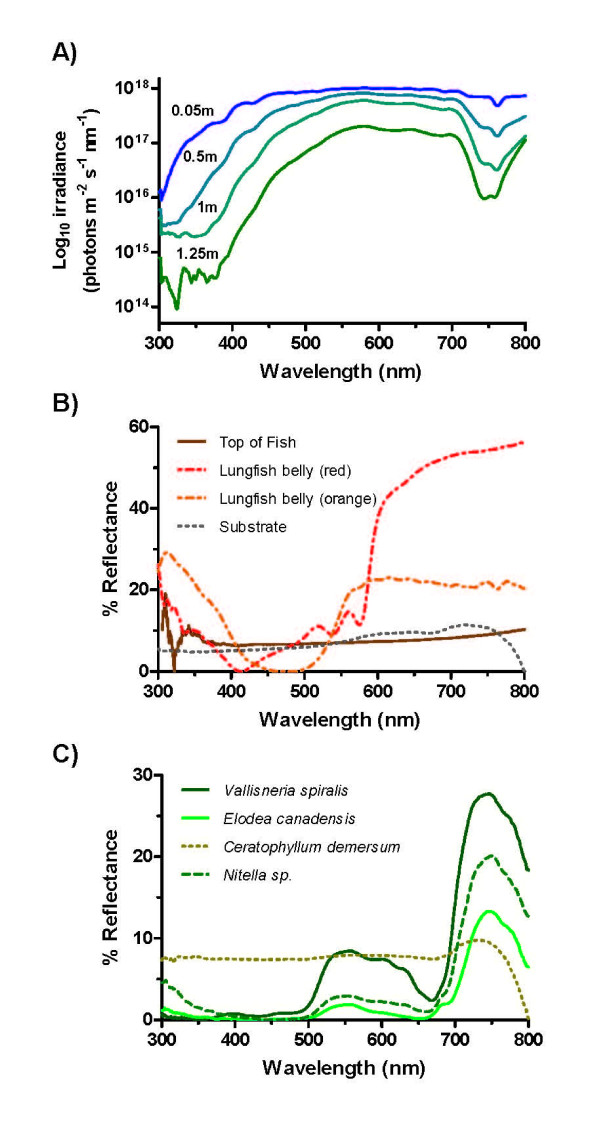
**Habitat irradiance and reflectance spectra of objects of potential importance to the visual ecology of lungfish**. **(A) **Downwelling spectral irradiance at increasing depths in a typical lungfish habitat, the Mary River, Queensland, Australia. Note the rapid attenuation of ultraviolet wavelengths with increasing depth compared to longer wavelengths. **(B) **Reflectance spectra of lungfish body colours compared to sandy riverbed substrate against which they might be viewed. **(C) **Reflectance spectra of typical macrophytes found in a typical lungfish habitat, some of which (e.g. *Vallisneria spiralis*) are eaten or used as spawning sites.

*G *and *G*^*0 *^denote the shape factor (essentially volume) of the object colour solid with and without spectral filters in the cone photoreceptors, respectively; *b *is the gross benefit due to the change in shape of the colour solid and is defined as *G*/*G*^0^; *d *is the factor describing the cost of the spectral filters due to increased signal noise. The overall (net) benefit is given as *r *= *b*/*d*. Discrimination thresholds are assumed to be limited by noise in receptor channels arising from fluctuations in the number of absorbed photons, i.e. shot noise under dim-light conditions. The benefit of spectral filters should be greater in brighter conditions; for details see Methods and [25]. Because at a depth of 1.25 m UV light is not present in significant amounts (Fig. 4A), the UV receptor was not used in calculations for the juvenile lungfish at this depth. The overall benefit to colour discrimination of the spectral filters in dim light is a factor of about 1.3 and is similar between juvenile and adults and between different habitat depths.

Calculations made with real habitat irradiance spectra measured at the surface of the water and also at 1.25 m depth (Fig. 4A) show that, even in dim light when fluctuations in the number of absorbed quanta (i.e. shot noise) limit visual performance, the yellow ellipsoidal/paraboloidal pigments and red oil droplet filters should improve the ability of juvenile and adult lungfish to discriminate colours by a factor of about 1.3 (Table 2). Although not modelled here, the improvements to colour vision should be more prominent in bright light, when noise in the photoreceptor channels is no longer determined by shot noise [25].

**Figure 5:**
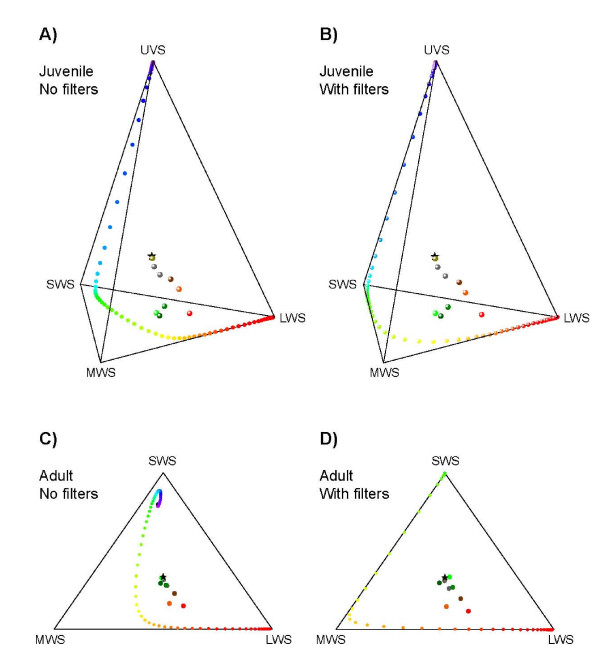
**Chromaticity diagrams of lungfish cone photoreceptor colour space**. Calculations were made for juvenile (**A, B**) and adult (**C, D**) lungfish, both with (**A, C**) and without (**B, D**) the filtering effects of the yellow ellipsoidal and red oil droplet spectral filters in the MWS and LWS cones. The black star in each diagram is the achromatic point. Large spheres/circles are the body colours (brown, orange, red), rocks and sand substrate (grey) and macrophytes (green) shown in Figs. 4B, C. Small circles represent monochromatic loci at 5nm intervals across the lungfish-visible spectrum. Note the increased separation of the object reflectance spectra and the displacement of the monochromatic loci towards the vertices in both juveniles (**B**) and adults (**D**) with spectral filters, relative to the situation without the effects of spectral filters. See Fig. 2 for explanation of abbreviations.

Reflectance spectra from objects of likely behavioural relevance to lungfish, such as macrophytes, rocks, sandy substrate and lungfish body colours (Figs. 4B, C), were plotted in the trichromatic colour space ('Maxwell triangle') of the adult and the tetrachromatic colour space (colour tetrahedron) of the juvenile (Figs. 5A–D). For both juvenile and adult lungfish, receptor colour spaces were calculated with and without the spectral filtering effects of red oil droplets and yellow ellipsoid/paraboloid pigments. In each case, the colour loci of theoretical monochromatic stimuli, which define the extent of the colour space, were also plotted at 5 nm intervals across the spectrum. In both age groups, the presence of spectral filters in the cone photoreceptors displaces the monochromatic loci towards the vertices and expands the available colour space. This is analogous to the expansion of the volume of the object-colour solid shown in Fig. 3. Most of the 'real' objects (especially some weeds, sand and rock) have relatively low absolute reflectances and/or reflectances that vary relatively little across the spectrum. Consequently, these data plot close to the achromatic point at the centre of the colour space (black star in Figs. 5A–D). Nevertheless, the loci of these real spectra are more widely dispersed (i.e. more easily discriminated) than they otherwise would be if the lungfish lacked intracellular spectral filters in the MWS and LWS single cones. Although these results are largely qualitative (given that important visual performance parameters, such as behaviourally determined discrimination thresholds for the lungfish, are not available) the modelling suggests that the presence of coloured filters has the potential to improve the colour discrimination ability of both juvenile and adult lungfish, as is thought to be the case for birds and turtles [21,25].

## Discussion

Our results show that the Australian lungfish *Neoceratodus forsteri *has the potential for a complex colour vision system based on either trichromacy (adults) or tetrachromacy (juveniles). They also show that, by reducing the overlap of the spectral sensitivities of adjacent spectral cone types, short-wavelength-absorbing spectral filters in certain cone types improve colour discrimination ability, despite the unavoidable reduction in total photon catch. Previous studies have highlighted the theoretical advantages of retinal spectral filters for colour discrimination in terrestrial birds and semi-aquatic freshwater turtles [21,25], but this is the first study to demonstrate the potential benefits to a purely aquatic animal, even in dim light where shot noise would limit visual performance. It is possible that the increase in signal noise due to absorption of light by the coloured filters is partially offset by the comparatively large diameter of the lungfish's photoreceptor inner and outer segments (Table 1), which would capture more light than the narrower photoreceptors typically found in the retinas of, for example, teleost fish living in a similar habitat [e.g. [26]]. Similar to *Neoceratodus *in this regard is the upstream migrant of the southern hemisphere lamprey, *Geotria australis*, which also has relatively large photoreceptors compared to other aquatic vertebrates and has dense, granular yellow pigmentation in the ellipsoid region of certain cone types [27].

It remains to be determined whether or not the red cone oil droplets in lungfish and tetrapods are derived from a similar structure in a common ancestor. To date, lungfish are the only truly aquatic animals to have red oil droplets. Some ray finned (actinopterygian) fish, such as the killifish *Fundulus heteroclitus *[28], four-eyed fish *Anableps anableps *[29], sturgeon *Acipenser shrenckii *[30] and the bowfin *Amia calva *[31] have oil droplet-like structures in their cone ellipsoid region that are either non-pigmented or, in the case of *Anableps*, contain a pigment resembling reduced cytochrome c that absorbs strongly below 450 nm [29]. Amongst the Amphibia, which diverged from the main vertebrate stock some 30 million years after the Dipnoi [ca. 360 MYA, [32]], only some frogs (Anura) have photoreceptor oil droplets. In the green frog, *Rana clamitans*, the oil droplets are described as "colourless" [33], although they are reported to have considerable absorption of wavelengths below about 500 nm (and, theoretically, should appear pale yellow). The strawberry poison frog, *Dendrobates pumilio*, is a diurnal species and its oil droplets are truly colourless, having negligible absorption across the spectrum [34]. In fact, of the extant 'higher' vertebrate taxa, red oil droplets are only found in birds and turtles. Although red oil droplets are retained in, and presumably useful to, both marine [35] and freshwater turtles [36], the fact that these groups are secondarily aquatic [37] suggests that either they evolved independently in terrestrial reptiles, or were lost from the lineage that gave rise to the amphibians.

The red oil droplets of lungfish LWS cone photoreceptors resemble closely those of some terrestrial vertebrates and, therefore, provide circumstantial support for the findings from molecular genetic studies that lungfishes are more closely related to the ancestors of the tetrapods than other extant sarcopterygian fish [e.g. [4]], which lack coloured oil droplets. However, the considerable plasticity of the vertebrate visual system requires that we must exercise caution in making such inferences, as other members of the Sarcopterygii, such as the ancestors of the coelacanth, may have lost coloured oil droplets/spectral filters following their divergence from the tetrapod lineage.

There may be other reasons for having yellow and red spectral filters besides colour vision. These include the absorption of scattered short-wavelength light, which would improve visual contrast detection by the MWS and LWS single cones in the same way that UV-blocking 'haze' or yellow filters improve contrast in photography [31]. Ultraviolet-absorbing filters may also protect the retina against damaging short-wavelength light [38]. However, the presence of a dedicated UVS cone type in the retina of the juvenile lungfish suggests that ultraviolet wavelengths are important in lungfish behavioural ecology, at least during specific lifecycle stages, and that they have strategies to cope with any UV-induced retinal damage.

UVS cones were not found in the retina of the adult lungfish and, although they may have been present in such low numbers that they were overlooked during the microspectrophotometric study, the greatly increased absorption of short wavelengths by the ocular media of adults, compared to those of juveniles (Fig. 2C), suggests that the amount of ultraviolet light reaching the adult retina is relatively very low and that ultraviolet sensitivity may be of little importance to the adults. Hatchling lungfish are predominantly carnivorous, feeding on worms and small crustaceans [7]. As their dentition develops, from sharp cones to crushing molariform tooth plates, their diet changes to include snails and other molluscs, larger crustaceans and, eventually, considerable amounts of plant material, such as the macrophyte *Vallisneria spiralis *[7]. UV sensitivity may be important to hatchling lungfish that prey on *Daphnia *or other planktonic prey; zooplankton often absorb or scatter UV wavelengths differently to the surrounding water and this enhancement in visual contrast may be used by predators for initial detection of, or discrimination between, preferred prey species [39,40]. However, ultraviolet sensitivity does not appear to be essential for zooplanktivory, at least in certain habitats: seahorses and pipefish (Sygnathidae) are highly specialised visual zooplanktivores that apparently lack UV visual pigments and have ocular media that block wavelengths below 400 nm [41]. Moreover, the juvenile lungfish examined in this study that were found to have UVS cones would probably no longer feed predominantly on planktonic prey.

The apparent loss of UVS cones from the adult lungfish retina reflects the situation observed in some teleost fishes that also show ontogenetic shifts in preferred habitat. For example, surface-feeding juvenile yellow perch (*Perca flavescens*) have small single cones containing a 400 nm λ_max _visual pigment that are lost entirely from the retinal mosaic as the fish matures and becomes demersal [39]. Brown trout (*Salmo trutta*) and rainbow trout (*Salmo gairdneri*) also lose their UVS cones (λ_max _340–360 nm) during development [42,43] and, like the lungfish, may revert from a potentially tetrachromatic colour vision system based on 4 cone visual pigments as juveniles to a trichromatic system as adults. Ultraviolet light is absorbed rapidly with increasing depth, especially in fresh water ([44], and see Fig. 4A). Perhaps unsurprisingly, older trout lacking UVS cones tend to live in deeper water than UVS cone-bearing juveniles [42], an ontogenetic trend reflected in the behaviour of *Neoceratodus *[7,17] that may explain the absence of UVS cones in the adult retina.

At the other end of the spectrum, the peak sensitivity of the LWS cone (656 nm) in adult *Neoceratodus *is, to our knowledge, the most long-wavelength-shifted of any vertebrate photoreceptor studied to date. A shift in spectral sensitivity towards longer wavelengths may be adaptive for animals living in nutrient-laden 'green' freshwater habitats, which are relatively richer in red light compared to most marine environments [23]. Habitat irradiance may, at least in part, explain the use of 3,4-didehydroretinal as the visual pigment chromophore in many freshwater fish [45], lampreys [27,46] and also *Neoceratodus*. This chromophore creates visual pigments with long-wavelength-shifted λ_max _compared to the same visual pigment (opsin) protein when conjugated with 11-*cis *retinal, and extends the extreme long wavelength limit of vertebrate visual pigment λ_max _(without additional spectral filters) from about 575 to 625 nm [47,48]. Moreover, taken together with the peak sensitivity of the MWS cones (584 nm), which is long-wavelength-shifted from the location of the visual pigment λ_max _by the yellow ellipsoidal/paraboloidal pigmentation, the enhanced long-wavelength sensitivity of *Neoceratodus *suggests that the discrimination of objects differing in their reflectance at long wavelengths is very important. In this regard, it is interesting to note that many of the macrophytes, including those (e.g. *Vallisneria spiralis*) that lungfish use for food, shelter or spawning [7], have reflectance spectra that differ predominantly at wavelengths above 500 nm (Fig. 4C); our modelling (Figs. 5A–D) shows how the red and yellow spectral filters potentially improve the ability of lungfish to discriminate between these macrophytes visually.

The spectral sensitivity of the rod photoreceptor of *Neoceratodus *is also shifted considerably towards longer wavelengths, having a visual pigment with a λ_max _at 540 nm. This spectral shift is mostly the result of the use of 3,4-didehydroretinal rather than 11-*cis *retinal as the chromophore and is similar to the situation observed in the 3,4-didehydroretinal-based rod visual pigments of teleost fish such as the rudd *Scardinius erythrophthalmus *(λ_max _540 nm) [49] and the European perch *Perca fluviatilis *(λ_max _540 nm) [45]. Like *Neoceratodus*, these fishes often inhabit green fresh water bodies that are relatively richer in long wavelengths of light.

The enhanced long-wavelength-sensitivity of the photopic visual system of *Neoceratodus *may also play a role in the assessment of body colouration in conspecifics. *Neoceratodus *are strongly countershaded (Fig. 1), having an olive-green to dark brown colouration on the dorsal surface and a pale underside that varies from light orange to pinkish red in adults (Fig. 4B). Juveniles are generally lighter in colour. The pink belly colour is apparently brighter in the breeding season, particularly in males [7], and the assessment of body colouration may be involved in mate choice, although this has yet to be investigated behaviourally.

## Conclusion

The lungfish has a complex retina with a single type of rod and either 3 (adults) or 4 (juveniles) cone photoreceptor types. Some of the cones possess coloured intracellular filters that tune the spectral sensitivity functions of the photoreceptors, enhancing the ability to discriminate between objects on the basis of colour, including those of ecological significance. In this respect, the visual system of the lungfish is much more like that of a turtle or a bird than an amphibian or a fish. The close phylogenetic relationship of the lungfish to the ancestors of the tetrapods, suggests that many of the ocular characteristics seen in extant terrestrial vertebrates may have evolved in water prior to the transition onto land.

## Methods

### Microspectrophotometry of retinal photoreceptors

Two adult lungfish (total length, TL, 105 and 110 cm) were caught from the Enogerra Reservoir, Queensland, Australia. Three captive-bred juvenile lungfish (TL 24, 28 and 32 cm) were donated by Prof. Jean Joss of Macquarie University, Australia. Animals were kept in large freshwater aquaria at the University of Queensland under a 12:12 light:dark cycle and fed a diet of commercial fish food. Animals were dark-adapted overnight, anaesthetised with benzocaine (Sigma-Aldrich Inc, St. Louis, MO, USA; pre-dissolved in acetone, dose 50 g l^-1^) and euthanased by spinal cord transection followed by pithing. All procedures were approved by the University of Queensland Animal Ethics Committee.

Eyes were removed under dim red light and all subsequent procedures were conducted under infra-red illumination with the aid of a dissecting microscope fitted with an infra-red image converter (ElectroViewer Series 7215, Electrophysics Corporation, Fairfield, NJ, USA). The retina was removed from the eye and placed in physiological saline [50]. Small pieces of retina (ca. 1 mm^2^) were dissected away and mounted on a 24 × 60 mm No. 1 glass coverslip in a drop of physiological saline containing 10% dextran (MW 282,000; Sigma-Aldrich Inc.). The retina was gently teased apart using mounted needles and the preparation covered with a 22 × 22 mm No. 0 glass coverslip. The edges of the top coverslip were sealed with nail varnish to prevent dehydration and movement of the retinal tissue.

Transverse absorption spectra (330–800 nm) of the inner and outer segments of retinal photoreceptors were made using a single-beam, wavelength-scanning, computer-controlled microspectrophotometer described in detail elsewhere [51,52]. Briefly, a sample scan was made by aligning the measuring beam of the microspectrophotometer within the photoreceptor and recording the amount of light transmitted at each wavelength. A baseline scan was then made of a cell-free area of the preparation adjacent to the photoreceptor and subtracted from the sample scan to create a 'pre-bleach' spectrum. Outer segments were bleached for 3 minutes with full spectrum 'white' light from the monochromator and identical sample and baseline scans were made to create a 'post-bleach' spectrum. The post-bleach spectrum was subtracted from the pre-bleach spectrum to generate a difference spectrum. Spectra were converted to either absorbance (outer segments) or absorptance (inner segments) and analysed as described elsewhere [22,53-55]. Individual spectra that satisfied established selection criteria [56,57] were retained for further analysis.

### Spectral transmittance of the ocular media

The spectral transmittance (300–900 nm) of the ocular media was measured in eyes taken from one of the juvenile and one of the adult lungfish used for microspectrophotometric analysis. In the adult, the cornea and lens were measured separately and the transmittance spectra combined. In the juvenile, a portion of the sclera at the back of the eye directly opposite the pupil was removed and the ocular media (including aqueous and vitreous humours) measured *in toto*. In each case, light from an Ocean Optics PX-2 Pulse Xenon Lamp (Ocean Optics, Dunedin, FL, USA) was delivered to the rear of the eye/lens/cornea using a 1000 μm diameter quartz fibre optic and transmitted light collected by a 50 μm diameter quartz fibre optic connected to an Ocean Optics S2000 spectroradiometer [58]. Transmittance spectra were smoothed with an 11-nm unweighted running average and normalized for display.

### Environmental light and spectral reflectance of biologically relevant colours

Downwelling irradiance spectra (300–800 nm) were measured using an Ocean Optics S2000 spectroradiometer in a typical *Neoceratodus *habitat: at the entrance to Chinaman's Creek in the Mary River, near Tiaro, Queensland, at midday on the 3^rd ^October 2003 (near the end of the dry season). Measurements were made 2 m out from the riverbank with readings taken at the surface and at various depths up to 1.25 m, as described elsewhere [52,59]. Water depth in the creek ranged to approximately 3 m. The water was green to our eyes but of relatively low turbidity. The substrate was mostly mud and sand, with sparse weed and log cover.

Spectral reflectance measurements (300–800 nm) of objects of potential biological relevance to *Neoceratodus *were made using an S2000 spectrometer, as described previously [60]. Measurements were made from stones, mud, sand and several macrophytes: *Vallisneria spiralis*, *Nitella sp*., *Ceratophyllum demersum *and *Elodea canadensis *taken from the Brisbane River at College's Crossing, Ipswich, Queensland, where lungfish are known to spawn (Ann Kemp, pers. comm.). Reflectance measurements were also made of the scales on the dorsal surface and two different areas of the ventral surface (one pink and one orange to the human eye) of an adult lungfish (TL 110 cm). All reflectance spectra were measured relative to a white standard (Spectralon; > 99% reflectance 400–1500 nm; Labsphere, North Sutton, NH, USA) and were smoothed with an 11-nm unweighted running average.

### Modelling spectral sensitivity, colour discrimination ability and receptor space

The quantal spectral sensitivity of each cone type in both juvenile and adult lungfish was calculated as the product of outer segment axial absorptance, oil droplet or ellipsoidal/paraboloidal pigment axial transmittance and ocular media transmittance [61]. The photoreceptor dimensions necessary for these calculations – which correct for the fact that we measured transverse rather than axial absorptance – were determined from images of the retina, projected onto a television screen during the microspectrophotometric experiments, using a calibrate acetate overlay. The specific absorbance of the visual pigment was 0.014 μm^-1^. Spectral sensitivity calculations were also made for the theoretical case where coloured filters are lacking from the cone inner segments, for comparison in the modelling of visual performance described below.

To determine the theoretical visual benefit of spectral filters (oil droplets and ellipsoidal pigments) within the inner segment of lungfish photoreceptors, we first used the 'object-colour solid' method of Vorobyev [24,25]. Briefly, a colour can be represented in the visual system of an observer as a point in *n*-dimensional colour space, where *n *is the number of photoreceptor types involved in colour vision. All theoretically possible surface colours occupy a part of that colour space, and together form a conceptual shape known as an object-colour solid. Each colour locus within the colour space is surrounded by an elementary volume containing very similar colours from which it is indistinguishable. The number of colours that can be discriminated by a given visual system is defined as the total number of these elementary volumes contained within the object-colour solid.

When fluctuations in the number of quanta (photons) absorbed by the cones determines colour discrimination thresholds, this elementary volume is proportional to the product of the square roots of the number of quanta absorbed by each receptor channel per integration time and per receptive field [24]. This allows us to calculate the benefit of spectral filtering independently of the intensity of ambient light. The overall (net) benefit (*r*) is given as *r *= *b*/*d*, where *b *is the (gross) benefit due to the change in shape of the object-colour solid and *d *is the factor describing the cost of the spectral filters due to the increased noise resulting from reduced transmission of photons to the visual pigment; *b *= *G*/*G*^0^, where *G *and *G*^0 ^denote the shape factor of the object colour solid with and without oil droplets, respectively [24]. In bright light, neural noise rather then quantum noise sets the threshold for colour discrimination and, theoretically, the benefit of oil droplets increases as the illumination becomes brighter [25]. The benefit of filtering in bright light can be calculated if the magnitude of this neural noise in the different receptor channels (i.e. Weber fraction) is known [24]; because the magnitude of neural noise in *Neoceratodus *is not known we do not present the data for bright light illumination. However, for the limiting case of dim light illumination, both the cost and benefit of coloured spectral filters can be calculated using microspectrophotometric data alone [25].

We also modelled the cone photoreceptor 'colour space' of *Neoceratodus *to investigate the appearance of real, behaviourally relevant objects found in their environment. While the object-colour solid method allows us to draw conclusions based on all possible colours and examine the ultimate limits of the lungfish colour vision system, receptor space modelling allows us to address more ecologically driven questions, such as the ability to discriminate between different types of vegetation and detect subtle differences in conspecific body colouration.

The calculation of receptor spaces (and their associated chromaticity diagrams) is described in detail by Kelber et al. [62]. Briefly, a visual system with *n *receptors is represented as an *n*-dimensional receptor space, the co-ordinate axes of which correspond to the relative photon catches of each cone photoreceptor type. Assuming that the colour-opponent mechanisms responsible for colour discrimination in the lungfish retina signal only chromatic (colour) differences between an object and the background against which it is viewed (a reasonable assumption for animals with duplex visual systems under photopic conditions), achromatic (intensity) information can be disregarded [63]. Receptor space can then be displayed as a chromaticity diagram with *n*-1 dimensions. In such a plot, pure excitation of any one cone type is represented by a locus at the relevant vertex; colours that excite more than one cone type are plotted according to their relative excitation ratios. The end result is a series of points whose relative distance from each other provides an indication of the likely discriminability of the colours to the visual system under examination, based on hue alone.

Reflectance spectra of lungfish colours and other relevant objects, measured relative to a white standard as described above, were multiplied by the downwelling irradiance spectrum (habitat light) and then multiplied by the quantal spectral sensitivity of each cone type. The resultant photon catch for each cone type was then divided by the sum of the photon catches for all cone types (to give relative photon catch) and plotted on the chromaticity diagram. The modelling was performed for both the juvenile and adult lungfish visual system, with and without the filtering effect of the coloured intracellular filters taken into account. Because there are no measurements of photoreceptor channel noise (Weber fractions) in lungfishes, we were unable to quantify the physiological relevance of any changes in colour distances.

## Authors' contributions

NSH carried out the microspectrophotometric measurements; assisted with animal capture, habitat light spectra measurements and colour space modelling; and wrote the manuscript. HJB coordinated animal capture and made the measurements of ocular media transmittance, spectral reflectance of lungfish and other object colours and habitat light spectra with NJM. MV and NJM assisted with colour space modelling. SPC helped with the conception of the project and assistance in the field. All authors read, contributed to and approved the final manuscript.
